# Diabetic Patients with Severe Sepsis Admitted to Intensive Care Unit Do Not Fare Worse than Non-Diabetic Patients: A Nationwide Population-Based Cohort Study

**DOI:** 10.1371/journal.pone.0050729

**Published:** 2012-12-07

**Authors:** Cheng-Wei Chang, Victor C. Kok, Ta-Chien Tseng, Jorng-Tzong Horng, Chun-Eng Liu

**Affiliations:** 1 Department of Information Management, Hsing Wu University, New Taipei City, Taiwan; 2 Public Health and Clinical Informatics Research Group, Department of Biomedical Informatics, Asia University Taiwan, Taichung, Taiwan; 3 Division of Medical Oncology, Department of Internal Medicine, Kuang Tien General Hospital, Taichung, Taiwan; 4 Bioinformatics Center, National Cheng Kung University, Tainan, Taiwan; 5 Department of Computer Science and Information Engineering, National Central University, Chungli, Taiwan; 6 Division of Infectious Diseases, Department of Internal Medicine, Changhua Christian Hospital, Changhua, Taiwan; University of São Paulo School of Medicine, Brazil

## Abstract

**Background:**

We sought to examine whether type 2 diabetes increases the risk of acute organ dysfunction and of hospital mortality following severe sepsis that requires admission to an intensive care unit (ICU).

**Methods:**

Nationwide population-based retrospective cohort study of 16,497 subjects with severe sepsis who had been admitted for the first time to an ICU during the period of 1998–2008. A diabetic cohort (n = 4573) and a non-diabetic cohort (n = 11924) were then created. Relative risk (RR) of organ dysfunctions, length of hospital stay (LOS), 90-days hospital mortality, ICU resource utilization and hazard ratio (HR) of mortality adjusted for age, gender, Charlson-Deyo comorbidity index score, surgical condition and number of acute organ dysfunction, were compared across patients with severe sepsis with or without diabetes.

**Results:**

Diabetic patients with sepsis had a higher risk of developing acute kidney injury (RR, 1.54; 95% confidence interval (CI), 1.44–1.63) and were more likely to be undergoing hemodialysis (15.55% *vs.* 7.24%) in the ICU. However, the diabetic cohort had a lower risk of developing acute respiratory dysfunction (RR = 0.96, 0.94–0.97), hematological dysfunction (RR = 0.70, 0.56–0.89), and hepatic dysfunction (RR = 0.77, 0.63–0.93). In terms of adjusted HR for 90-days hospital mortality, the diabetic patients with severe sepsis did not fare significantly worse when afflicted with cardiovascular, respiratory, hepatic, renal and/or neurologic organ dysfunction and by numbers of organ dysfunction. There was no statistically significant difference in LOS between the two cohorts (median 17 *vs.* 16 days, interquartile range (IQR) 8–30 days, *p* = 0.11). Multiple logistic regression analysis to predict the occurrence of mortality shows that being diabetic was not a predictive factor with an odds ratio of 0.972, 95% CI 0.890–1.061, *p* = 0.5203.

**Interpretation:**

This large nationwide population-based cohort study suggests that diabetic patients do not fare worse than non-diabetic patients when suffering from severe sepsis that requires ICU admission.

## Introduction

Severe sepsis, defined as deregulation of the inflammatory response to a documented infection complicated by acute organ dysfunction, causes substantial healthcare burdens and is a leading cause of death. Sepsis complicated by acute organ dysfunction accounts for almost half of intensive care unit (ICU) resource utilization and is associated with a higher morbidity and mortality than sepsis without acute organ dysfunction [1], [2]. Globally the mortality rate of severe sepsis ranges from 28.6% to 49.6%[1], [3]. Various risk factors, such as advanced age, chronic alcoholism and an immunosuppressed state, impact negatively on the treatment outcome of severe sepsis [1], [2], [4], [5]. Nevertheless, there is still conflicting data on whether diabetes mellitus is a negative determinant in relation to the outcome of severe sepsis [6], [7], [8], [9].

Diabetes mellitus is considered an immunosuppressed state [10]. Diabetic patients are particularly prone to endothelial dysfunction during sepsis. A recent study has shown that E-selectin, a soluble leucocyte adhesion molecule, and sFLT-1, a VEGF receptor, are significantly increased in diabetic patients compared with patients without diabetes during the most severe stages of sepsis; this suggests that patients with diabetes show a more pronounced activation of some endothelial pathways during sepsis, particularly during severe sepsis [11], [12]. The presence of diabetes mellitus seems to affect the already compromised red blood cell deformability of septic patients, probably leading to a further impairment of microcirculatory functionality in these patients [13].

A review of the literature on the effect of diabetes in relation to the outcome of severe sepsis or infection shows inconsistent results [6], [7], [8], [9], [13], [14], [15], [16], [17], [18], [19]. Thus, the magnitude of the effect of diabetes on the risk of organ failure and hospital mortality among patients with severe sepsis remains an active question. Therefore we sought to examine whether type 2 diabetes increases the risk of organ dysfunction and the risk of death following severe sepsis that required admission to an intensive care unit (ICU) using a nationwide population-based retrospective prospective study design. Relative risk (RR) for various organ dysfunctions, the length of hospital stay (LOS), the 90-days hospital mortality, ICU resource utilization and the hazard ratio (HR) for mortality were compared among patients with severe sepsis with or without diabetes.

## Subjects and Methods

### Data Sources

This study was designed as a population-based retrospective prospective study using the Taiwan National Health Insurance Research Database (NHIRD). The Taiwan National Health Insurance (NHI) program started in March 1, 1995. As at 2007, 98.4% of Taiwan's population of 22.96 million individuals was enrolled in this program. The NHIRD contains a number of large computerized databases that include registration files and original data on claims' reimbursement; these are derived from the insurance system held by the Bureau of National Health Insurance. These databases are maintained by the National Health Research Institutes (NHRI), Taiwan and are provided to researchers for academic research purposes. The databases of the NHIRD consists of four main databases: the ambulatory expenditures by visit file, the details of ambulatory care orders file, the inpatient expenditures by admission file and the details of inpatient orders file. These data files are de-identified by scrambling the identification codes of both the individuals and medical facilities; information on individuals is then sent to the National Health Research Institutes and forms the original files of the NHIRD, which has become one of the largest and most comprehensive population-based databases in the world. This data is generally regarded as very accurate and complete [20], [21], [22], [23].

The present study data were retrieved from a one million randomly-sampled enrollee dataset from the mother NHIRD. This consisted of 1 million randomly selected subjects that represent about 4.5% of Taiwanese population from the entire NHI enrollee profile. There were no significant differences in age and sex between the 1 million random sampling dataset and the mother NHI research database [24].

We utilized the databases for patients' demographics including encrypted identification number, gender, date of birth and death, dates of admission and discharge, diagnostic data and procedures (up to five) and discharge status (recovered, died or transferred out). The diagnostic data included date of initial diagnosis, specific treatment items, date of medical treatment, the relevant International Classification of Diseases, Ninth Revision, Clinical Modification (ICD-9-CM) diagnosis codes (up to five), and drug codes.

### Ethics Statement

This study utilized the Taiwan National Health Insurance Research Database (NHIRD) which is provided to researchers for academic research purposes. The data files are de-identified by scrambling the identification codes of both the individuals and medical facilities. This study adhered to strict confidentiality guidelines that are in accordance with the regulations regarding personal electronic data protection. As the data files consist of unidentified secondary data, the study was exempted from a full review by the Institutional Review Board. Obtaining informed consent from the study population was not required due to the de-identified data files, the large size of the population and to the part of the population that is unattainable (deceased) by the time of the study.

### Case Selection and Definition

The cases of severe sepsis in this study were newly diagnosed ones that required admission to an intensive care unit (ICU). Severe sepsis was defined as documented infection, either bacterial or fungal, plus at least one acute organ dysfunction using criteria based on the International Classification of Diseases, Ninth Revision, Clinical Modification (*ICD-9-CM*). *ICD-9-CM* codes used to identify a bacterial or fungal Infection were adopted from a published work in the literature [1]. The types of acute organ dysfunction associated with the severe sepsis included acute respiratory organ dysfunction (*ICD-9-CM* 93.90, 96.04, 96.7, 518.81, 518.82, 518.85, 786.09, 799.1); acute cardiovascular organ dysfunction (*ICD-9-CM* 458.0, 458.8, 458.9, 785.5, 785.51, 785.59, 796.3); acute hematologic organ dysfunction (*ICD-9-CM* 286.2, 286.6, 286.9, 287.3–287.5, 790.92); acute hepatic organ dysfunction (*ICD-9-CM* 570, 572.2, 573.3, 573.4); acute kidney injury (*ICD-9-CM* 39.95, 580.x, 584.x, 586) and acute neurological organ dysfunction (*ICD-9-CM* 89.14, 293, 348.1, 348.3, 780.01, 780.09) [1], [2], [25], [26]. In order to fulfill the definition of newly diagnosed sepsis, subjects who had been afflicted with sepsis at any time in the preceding two years were excluded. The definition of diabetes mellitus was that the ICD-9-CM code for diabetes (250.X) was present in Outpatient File at least three times in the year before severe sepsis was diagnosed. This definition and accuracy of diabetes diagnosis were studied in Taiwan: the probability of accurate diagnosis of diabetes among patients with >/ = 4 outpatient visits was 99.16 times greater than that of patients with </ = 1 outpatient visit. The probability of accurate diagnosis in patients with >/ = 1 hospitalization was 5.26 times that of patients who had not been hospitalized [27], [28].

Any comorbid medical conditions were identified using their standard ICD-9-CM codes and were used to calculate cumulatively the established Charlson-Deyo comorbidity index for each individual. The Charlson-Deyo comorbidity index score, adapted from the Charlson index for use with ICD-9-CM coded administrative databases, contains 17 weighted categories related to chronic concomitant diseases and is able to predict the subsequent 1-year mortality among inpatients. Each category has a score between 1 and 6 points (1 point for myocardial infarction, congestive heart failure, peripheral vascular disease, cerebrovascular disease, dementia, chronic pulmonary disease, rheumatological disease, peptic ulcer disease, mild liver disease, and diabetes without organ damage; 2 points for diabetes with organ damage, hemiplegia or paraplegia, severe renal disease, any malignancy including leukemia and lymphoma; 3 points for severe liver disease; 6 points for metastatic solid tumor and HIV infection), and sum of these scores is regarded as a measure of the burden of comorbidity [29], [30]. To avoid double-counting, diabetes mellitus was excluded from the CCI score in the diabetic cohort.

Acute organ dysfunction caused by severe sepsis included respiratory dysfunction, cardiovascular dysfunction, renal dysfunction (acute kidney injury), hepatic dysfunction, neurological dysfunction, and hematological dysfunction. The source of infection refers to the anatomic site of the infection, which includes the respiratory tract, the genitourinary tract, the gastrointestinal tract, skin, soft tissue or bone, the central nervous system, the cardiovascular system and others. Surgical conditions were defined as when a patient has a major surgical procedure other than tracheostomy based on the ICD-9-CM procedure codes.

### Survival Data

**Figure 1:**
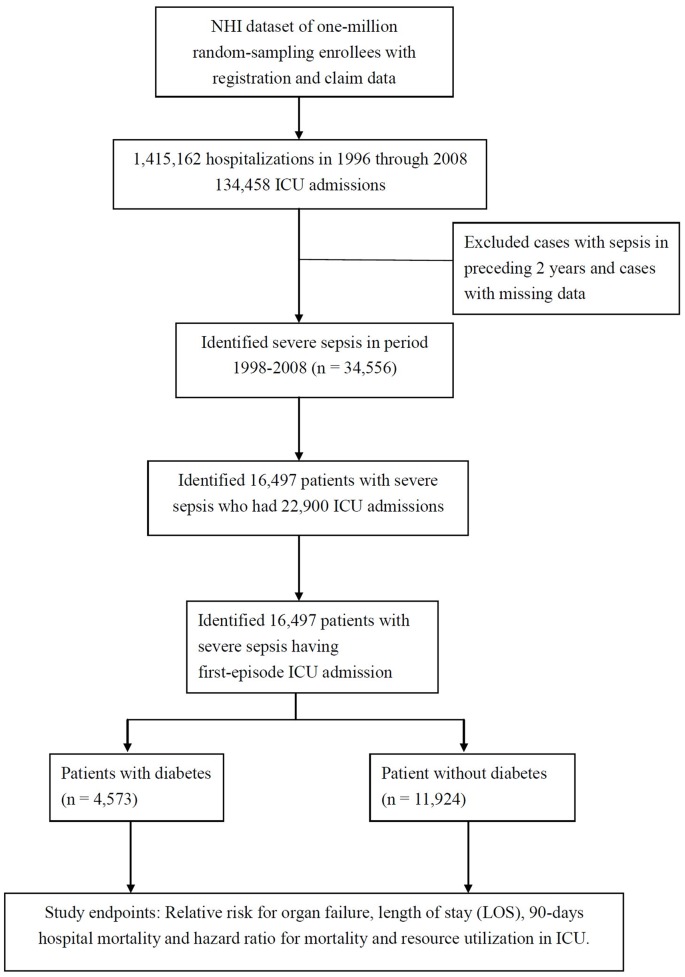
The Study Flowchart.

With respect to “discharge status”, the death of a patient was identified when either of two allocations were given in the record, that is either death or “discharged in terminally ill state” which is in Taiwan means that the patient is moribund or near to death. As depicted in the figure 1, the study endpoints for the cohorts studied here are the relative risk of organ dysfunction, the length of hospital stay (LOS), the 90-day hospital mortality and the hazard ratio for death. ICU resource utilization between the two groups was also compared.

### Statistical Analysis

The SAS statistical package (SAS System for Windows, version 8.2, SAS Institute, Cary, NC, USA) was used for pre-analysis data file merging and other data management before the statistical analysis. The statistical analysis was performed using SPSS software (version 17.0, SPSS Inc., Chicago, Illinois, USA). All statistical tests were two-sided. Values of *p*<0.05 were considered statistically significant. The risk of death due to sepsis was evaluated using Cox proportional hazards analysis. Hazard ratios with a 95% confidence interval were calculated.

The distribution of patients and disease characteristics, with or without ICU admission, were compared between patients with and without diabetes, and the differences were examined using the χ^2^ test and the t test, as well as the Mann–Whitney U test (also called the Wilcoxon rank-sum test), which is a non-parametric statistical hypothesis test for assessing whether one of two samples of independent observations tends to have larger values than the other. Categorical variables such as age, sex, surgical condition, underlying comorbidity, infection site, and hospital mortality are reported as percentages. Continuous variables, such as the Charlson-Deyo comorbidity index score and hospital length of stay, are reported as means and medians. The StatsDirect statistical software (StatsDirect Ltd, England, 2008) was used to provide relative risk (RR) values and Yate's corrected χ^2^ test computations.

The 90 days cumulative survival probabilities of unjustified mortality related to severe sepsis were estimated by Kaplan-Meier method. Survival curves among patients with different types of severe sepsis were created individually based on their diabetes status. The log-rank test was used to compare the significance of inequality with respect to the diabetes status curves. Cox proportional hazards models were performed for all types of the hazard ratios in relation to severe sepsis with diabetes; these were adjusted for sex, age, number of comorbid conditions, number of organ failures, and surgery status.

Our results were used to construct a multiple logistic regression model to predict the occurrence of mortality, Y, based upon the following five explanatory variables: diabetes, X_1_, gender, X_2_, age group, X_3_, Charlson-Deyo comorbidities index score, X_4_, and number of organ failure, X_5_. The 95% confidence interval (95% CI) for the estimated odds ratio (OR) was then calculated.

## Results

In the one-million representative sample of the National Health Insurance of Taiwan consisting complete corresponding registration and claim data, there were 1,415,162 hospitalizations from 1996 through 2008 among which there were 134,458 ICU admissions. We then identified 34,556 patients with severe sepsis during the period from 1998 to 2008 after excluding patients who had had sepsis in the preceding two years and cases with missing data. From this pool of patients, we identified 16,497 patients with severe sepsis who had been admitted to the ICU for the first time. These patients were then separated into a diabetes cohort (n = 4573) and a non-diabetic control group (n = 11924). Both groups of patients were then studied with respect to their characteristics, acute organ dysfunction, length of hospital stay (LOS), resource utilization in the ICU and followed up till discharge or death (Fig. 1).

### Demographic Characteristics

The mean age of the diabetic patients was 71.6 years, which is statistically significantly older than that of the non-diabetic patients, 67.6 years (*p*<0.0001). Male patients accounted for 53.84% of all diabetic patients whereas male patients account for 64.16% of the non-diabetic patients. In terms of co-morbidities, both groups showed no statistical difference in the Charlson-Deyo comorbidities index score (2.13 *vs.* 1.98, *p*>0.9999 Mann-Whitney U test). Both groups were balanced in terms of surgical conditions and having malignant neoplasm (cancer) (14.63% *vs.* 14.45%) (Table 1). Diabetic patients had more infections of the genitourinary tract (26.09% *vs.* 16.25%), gastrointestinal tract (34.53% *vs.* 32.58%), and skin, soft tissue and bone (19.07% *vs.* 12.00%) when their sites of infection were compared (Table 1).

**Table 1:** Demographic characteristics and clinical features of patients with severe sepsis in their first-episode ICU admission.

	Severe sepsis with first-episode ICU admission				*P*-value
	DM (n = 4573)		Non-DM (n = 11924)		
Age group\mean	71.63			67.57	<0.0001
<50	216	4.72%	2062	17.29%	
50–60	477	10.43%	1120	9.39%	
60–70	996	21.78%	1642	13.77%	
70–80	1663	36.37%	3333	27.95%	
>80	1221	26.70%	3767	31.59%	
Gender: male	2462	53.84%	7651	64.16%	<0.0001
Surgical condition	1243	27.18%	3172	26.60%	0.4554
Charlson-Deyo comorbidity index (CCI) score+	2.13		1.98		>0.9999++
CCI Low (0–2)	2775	60.68%	7824	65.62%	0.4++
CCI moderate (3–4)	1025	22.41%	2505	21.01%	
CCI high (≥5)	773	16.90%	1595	13.38%	
Malignant neoplasm	669	14.63%	1723	14.45%	0.7884
Number of infections by site					
Respiratory	1991	43.54%	5166	43.32%	0.806
Genitourinary	1193	26.09%	1938	16.25%	<0.0001
Skin, soft tissue, or bone	872	19.07%	1431	12.00%	<0.0001
Gastrointestinal	1579	34.53%	3885	32.58%	0.018
Central nervous system	47	1.03%	140	1.17%	0.4603
Cardiovascular	20	0.44%	71	0.60%	0.2417
Others	228	4.99%	451	3.78%	0.0005
		129.67%		109.71%	

DM: Diabetes Mellitus, CCI: Charlson-Deyo Comorbidity Index,

+ To avoid double-counting, diabetes mellitus was excluded from the CCI score in the diabetic cohort.

++ Mann-Whitney U test.

In this Taiwanese population-based study, a higher percentage of non-diabetic patients with severe sepsis who were admitted to an ICU were mechanically ventilated, 76.16% of diabetic patients *vs.* 78.83% of non-diabetic patients (*p* = 0.0002). As compared with the non-diabetic patients, diabetic patients more frequently underwent urinary catheterization (72.86% *vs.* 69.33%, *p*<0.0001) and hemodialysis (15.55% *p*. 7.24%, *p*<0.0001) and to lesser extent, percutaneous nephrostomy (0.59% *vs.* 0.35%, *p* = 0.0469). Whereas, non-diabetic patients in this cohort study were slightly more likely to have a chest tube insertion procedure (4.39% *vs.* 3.48%, *p* = 0.0098). These ICU resource utilizations may reflect that acute kidney injury requiring hemodialysis is more frequently observed in the diabetic patients with severe sepsis.

### Risk of Acute Organ Dysfunction(s) by Diabetes Status

Diabetic patients with severe sepsis who were admitted to an ICU had an increased relative risk (RR) of getting acute kidney injury (RR = 1.54, 95% CI, 1.44–1.63). Other than acute kidney injury, it is interesting to note that diabetic patients had a decreased relative risk of developing respiratory organ dysfunction (RR = 0.96, 95% CI, 0.94–0.97), hematological dysfunction, such as secondary thrombocytopenia, unspecified thrombocytopenia, other coagulation defects or defibrillation syndrome, (RR = 0.70, 95% CI, 0.56–0.89) and hepatic organ dysfunction (RR = 0.77, 95% CI, 0.63–0.93) (Table 2). The only increased RR is that of acute kidney injury. The rates are 27.14% for diabetic patients with sepsis and acute kidney injury as compared to 17.68% for the non-diabetic cohort (Table 2). Our results also show that 15.55% of diabetes group with severe sepsis underwent hemodialysis; this is much higher than the rate of 7.24% found for non-diabetic subjects (*p*<0.0001). However in terms of the HR for hospital mortality rate, the group with acute kidney injury was found to have a lower risk of 0.795 (0.691–0.914). After adjustment made for age group, gender, Charlson-Deyo comorbidity index, surgical condition and number of acute organ dysfunctions, the adjusted HR became non-significant (aHR = 0.871, 95% CI 0.754–1.005).

**Table 2:** Outcomes of acute organ dysfunction(s) and 90-days in-hospital mortality rate in patients with severe sepsis by diabetes mellitus status.

	Severe sepsis with ICU admission (N = 16497)				Relative risk (95% CI)
	With diabetes (n = 4573)		Without diabetes (n = 11924)		
Acute Organ Dysfunction					
Respiratory	3542	77.45%	9649	80.92%	0.96 (0.94–0.97)*
Cardiovascular	1309	28.62%	3488	29.25%	0.98 (0.93–1.03)
Hematologic	90	1.97%	333	2.79%	0.70 (0.56–0.89)*
Hepatic	126	2.76%	428	3.59%	0.77 (0.63–0.93)*
Kidney	1241	27.14%	2108	17.68%	1.54 (1.44–1.63)*
Neurological	173	3.78%	418	3.51%	1.08 (0.91–1.28)
Hospital Mortality	1034	22.61%	2694	22.59%	1.00 (0.94–1.07)

* statistically significant.

### 90-days in-hospital mortality and Cox proportional hazard model

**Figure 2:**
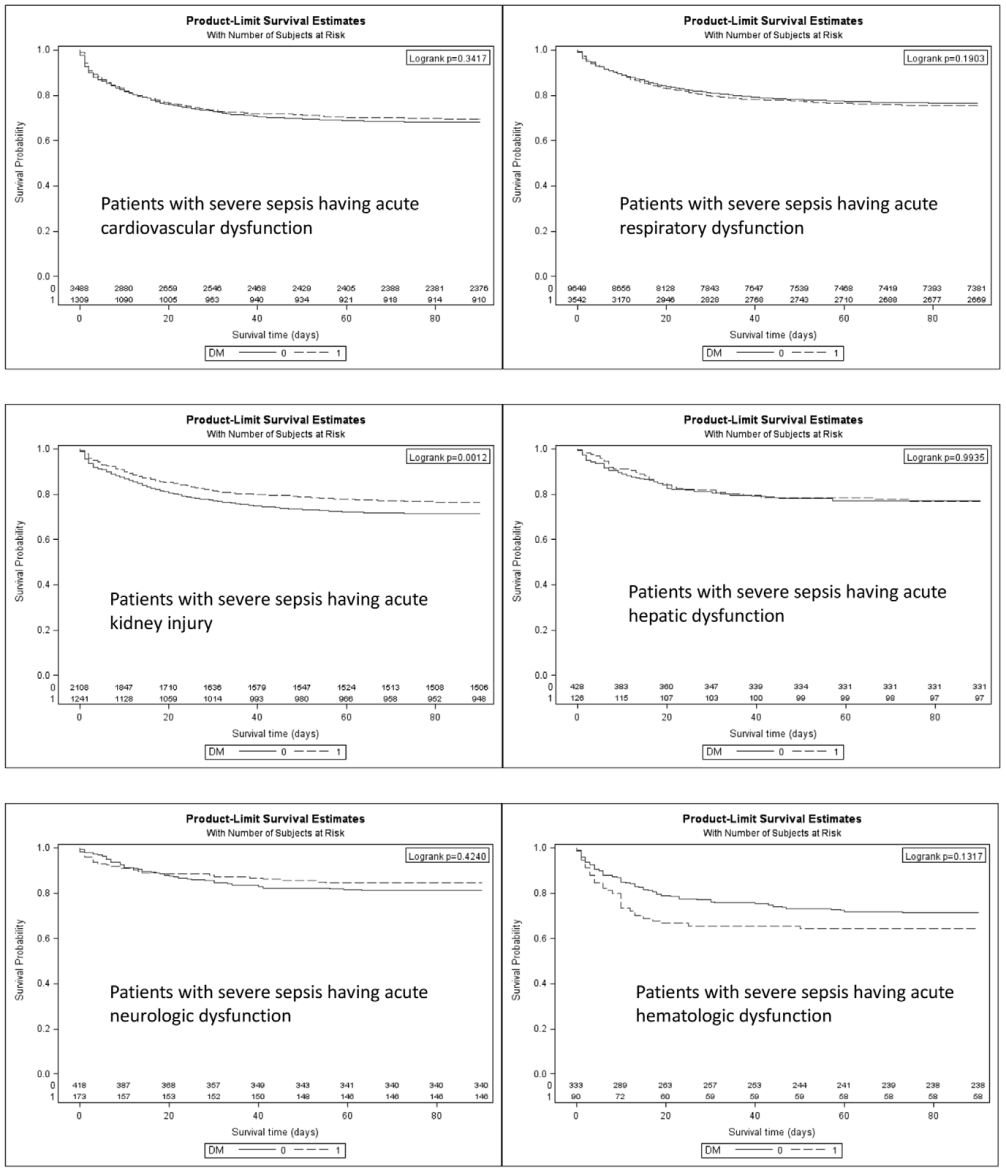
Kaplan-Meier survival curves in diabetic and non-diabetic patients with severe sepsis shown by type of acute organ dysfunction. Log-rank test was used for comparing their survival.

**Figure 3:**
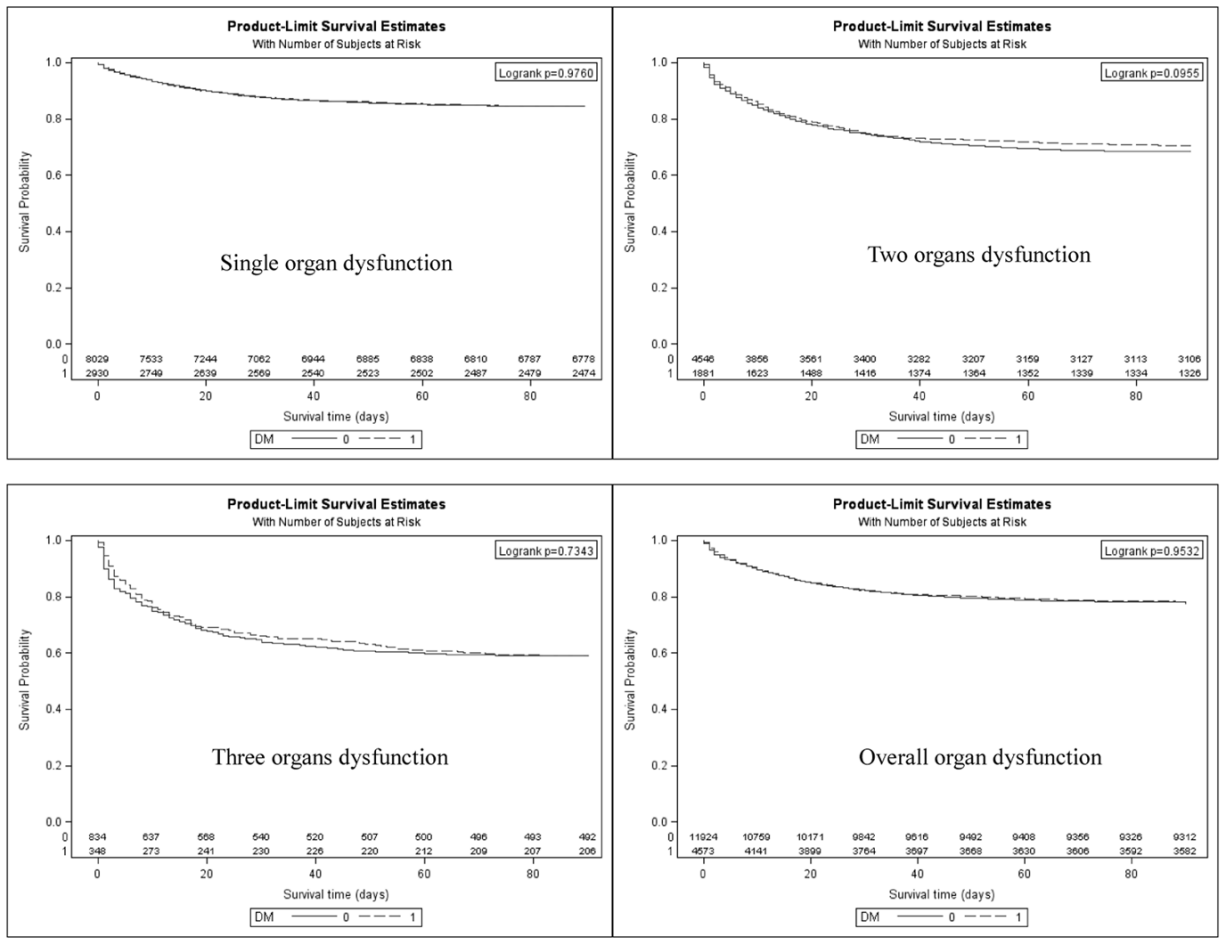
Kaplan-Meier survival curves in diabetic and non-diabetic patients with severe sepsis by number involvement of organ dysfunction. Log-rank test was used for comparing their survival.

Diabetic patients with severe sepsis who had acute organ dysfunction do not fare worse than non-diabetic patients in terms of hospital mortality rate, 22.61% *vs.* 22.59%, or relative risk = 1.00 (95% CI = 0.94–1.07) (Table 2). Patients with diabetes complicated with acute organ dysfunction across the board also do not fare worse as evidenced by the unadjusted hazard ratio (HR) (Table 3). After adjusting for age group, gender, Charlson-Deyo comorbidities index, surgical condition, and number of organ dysfunctions, the diabetic patients had a significantly lower HR for death (did not fare worse) depending on type of acute organ dysfunction (Fig. 2). These were an adjusted HR = 0.952 (95% CI, 0.848–1.067) for cardiovascular organ dysfunction, an adjusted HR = 1.020 (0.942–1.105) for respiratory organ dysfunction, an adjusted HR = 1.008 (0.654–1.552) for hepatic dysfunction, an adjusted HR = 0.871 (0.754–1.005) for acute kidney injury and an adjusted HR = 0.763 (0.473–1.231) for acute neurological dysfunction (Table 3, Figure 2). In terms of number of organ dysfunctions, the patients with diabetes also did not fare worse than non-diabetics with an adjusted HR for death of 0.979 (0.908–1.055) for the groups with overall organ dysfunction, an adjusted HR = 1.001 (0.898–1.117) for single organ dysfunction, an adjusted HR = 0.929 (0.825–1.045) for two-organ dysfunction and an adjusted HR = 1.086 (0.879–1.342) for groups with ≥3 organ dysfunction (Figure 3). As a whole, patients with diabetes who had complications due to acute organ dysfunction did not fare worse, with an adjusted HR of 0.979 (0.908–1.055), after an adjustment was made for age group, gender, Charlson-Deyo comorbidity index and number of organ dysfunctions (Table 3).

**Table 3:** Cox proportional hazard model assessing the effect of diabetes on 90-days hospital mortality in patients with severe sepsis having at least one organ dysfunction in their first-episode ICU admission.

Organ failure	90-days hospital mortality rate (%)		Hazard Ratio (95% CI)	Adjusted^a^ Hazard Ratio (95% CI)
	DM	non-DM		
Cardiovascular	30.48%	31.88%	0.946 (0.844–1.061)	0.952 (0.848–1.067)
Respiratory	24.65%	23.51%	1.054 (0.975–1.140)	1.020 (0.942–1.105)
Hematologic	35.56%	28.53%	1.357 (0.909–2.025)	1.558 (1.010–2.402)*
Hepatic	23.01%	22.66%	1.002 (0.662–1.517)	1.008 (0.654–1.552)
Kidney	23.61%	28.56%	0.795 (0.691–0.914)**	0.871 (0.754–1.005)
Neurologic	15.61%	18.66%	0.837 (0.540–1.297)	0.763 (0.473–1.231)
Overall organ dysfunction	21.67%	21.91%	0.986 (0.917–1.061)	0.979 (0.908–1.055)
Single organ dysfunction	15.56%	15.58%	0.998 (0.897–1.111)	1.001 (0.898–1.117)
Two organ dysfunction	30.01%	33.37%	0.879 (0.784–0.986) *	0.929 (0.825–1.045)
≥3 organ dysfunction	44.00%	42.07%	1.035 (0.843–1.270)	1.086 (0.879–1.342)

CI: confidence interval; DM: diabetes mellitus; ICU: intensive care unit.

a Adjustment was made for age group, gender, Charlson-Deyo co-morbidity index, surgical condition, number of acute organ dysfunction. To avoid double-counting, diabetes mellitus was excluded from the CCI score in the diabetic cohort.

* *P*-value<0.05;

** *P*-value<0.01.

### Length of stay (LOS)

Both the mean and median days of stay per ICU hospitalization and interquartile range (IQR) of the LOS of the two cohorts showed no statistically significant difference (*p* = 0.11) (Table 4). The mean LOS was approximately 24 days in both cohorts and the median LOS was 17 days for diabetic patients versus 16 days for non-diabetic patients.

**Table 4:** Total days of stay per ICU hospitalization.

	Severe Sepsis with Diabetes	Severe Sepsis without Diabetes	Difference	*Ρ*-value*
Mean	23.85	23.72		
Median	17.00	16.00	1	0.11
Standard deviation (SD)	33.52	44.93		
Interquartile Range (IQR)	8.0–30.0	8.0–30.0		

* Wilcoxon signed rank test.

### Multiple Logistic Regression Model to Predict Occurrence of Death

In a multiple logistic regression analysis to predict in-hospital death based on diabetes mellitus status, gender, age group, Charlson-Deyo comorbidity index in the low, moderate or high score groups, and number of organ dysfunctions, diabetes status does not result in an increased probability of death with an odds ratio (OR), 0.972 (95% CI = 0.890–1.061), *p* = 0.5203 (Table 5). The independent predictive factors for in-hospital mortality in this model are male sex, OR 1.299 (1.198–1.408), an older age group (60–70 years, OR 0.812 (0.698–0.954); older than 80, OR 1.292 (1.136–1.470)), a higher CCI score group (moderate (score 3–4), OR 1.614 (1.471–1.771); high (>/ = 5), OR 1.887 (1.699–2.096)), and having more than one organ dysfunction (two-organ-dysfunction, OR 2.533 (2.334–2.749); >/ = 3-organ-dysfuction, OR 3.995 (3.483–4.582)). However, the 60–70 year age group had a lower risk, namely an OR of 0.8 (0.698–0.945). Thus diabetes mellitus status does not predict patient mortality outcome based on the present multiple logistic regression analysis.

**Table 5:** Multiple logistic regression analysis to predict the occurrence of mortality based on diabetes, gender, age group, Charlson comorbidities index and number of organ failure.

	Coefficient (b)	Odds ratio	95% CI	P value
DM vs. non-DM	−0.0288	0.972	0.890–1.061	0.5203
Male sex	0.2614	1.299	1.198–1.408	<.0001
Age group				
<50	Reference			
50–60	0.00308	1.003	0.850–1.184	0.9709
60–70	−0.2081	0.812	0.698–0.945	0.0070
70–80	−0.0208	0.979	0.859–1.117	0.7568
>80	0.2561	1.292	1.136–1.470	<.0001
CCI				
Low(0–2)	Reference			
Moderate(3–4)	0.4786	1.614	1.471–1.771	<.0001
High(> = 5)	0.6351	1.887	1.699–2.096	<.0001
Organ Dysfunction				
1	Reference			
2	0.9293	2.533	2.334–2.749	<.0001
≥3	1.3850	3.995	3.483–4.582	<.0001

95% CI: 95% confidence interval; DM: diabetes mellitus; CCI: Charlson-Deyo comorbidities index score. To avoid double-counting, diabetes mellitus was excluded from the CCI score in the diabetic cohort.

## Discussion

This is the first report utilizing a nationwide population-based follow-up study design that explores the issue of whether diabetes has negative impact on the outcome of severe sepsis. Previous studies examining this relationship have narrowed down certain specific aspects, such as concentrating on certain types of single organ failure or using a specific care setting such as the Emergency Room [6], [8], [16], [17], [19], [31]. Morbidity from diabetes is a consequence of both microvascular disease (retinopathy, nephropathy, and neuropathy) and macrovascular disease (atherosclerosis), and therefore organ dysfunctions are often inter-related. In these circumstances a population-based study is likely to be more convincing as it will examine more types of organ dysfunctions following the occurrence of severe sepsis. Table 6 lists all published research in this area that to our knowledge compares the outcomes, particularly mortality rate, between diabetics and non-diabetics with sepsis or severe infection including pneumonia and enterobacterial bacteremia (Table 6).

**Table 6:** Previous published works comparing the outcomes particularly the mortality rate between diabetics and nondiabetics with sepsis or severe infection.

Authors	Published year	Mortality rate (DM vs. non-DM)	Study Settings
Carton JA et al.	1992	Overall mortality and bacteremia-related mortality were similar in both groups.	Prospective study of all adult pts with bacteremia admitted to a large Spanish teaching hospital
Kornum JB et al.	2007	Mortality among diabetic pts was greater than that among other pts: 19.9 vs. 15.1% after 30 days and 27.0 vs. 21.6% after 90 days, corresponding to adjusted 30- and 90-day MRRs of 1.16 (95% CI 1.07–1.27) and 1.10 (1.02–1.18).	Population-based cohort study of adults with a first-time hospitalization for pneumonia
Moutzouri AG et al	2008	The mortality in non-diabetic septic pts was 22.5% and in septic diabetics was 34.3%.	40 pts suffering from severe sepsis, 12 pts suffering from diabetes and 24 diabetic pts with severe sepsis were enrolled.
Stoeckle M et al	2008	In-hospital mortality rate was similar in the two groups (18% vs. 14%).	During a 4-year period 71 diabetic and 252 non-diabetics with bloodstream infection were included.
Esper AM et al.	2009	People with DM were less likely to develop acute respiratory failure (9% vs. 14%, p<0.05) and more likely to develop acute renal failure (13% vs. 7%, p<0.05).	Using the National Hospital Discharge Survey US, sepsis cases from 1979 to 2003 were integrated with DM prevalence from the CDC Diabetes Surveillance System.
Kofteridis DP et al	2009	People with DM had longer fever (median 4.5 vs 2.5 days; P<.001), longer hospitalization (median 10 vs 7 days; P<.001), and greater mortality (12.5% vs 2.5%; P<.01) than controls.	88 pts aged 65 and older with DM and 118 controls without DM, matched for age and sex, hospitalized with acute pyelonephritis
Peralta G et al.	2009	Mortality among diabetic and non-diabetic pts was not different [7.2% vs. 8.2%, RR 1.13; 95% CI (0.67–1.9); p = 0.39].	Retrospective cohort study to investigate prognosis in pts with Enterobacteriaceae bacteremia.
Stegenga ME et al	2010	Mortality was equal in diabetic and nondiabetic pts (31.4% vs. 30.5% after 28 days).	Retrospective analysis of a previously published study.
Schuetz P et al.	2011	The mortality rate was 4.3% (95% CI 3.9% to 4.8%) and similar in diabetic and nondiabetic pts (4.1% versus 4.4%; absolute risk difference 0.4%; 95% CI −0.7% to 1.4%).	3 independent, observational, prospective cohorts from Emergency Department pts with sepsis from 2 large US tertiary care centers
Chang C et al	This study	Diabetic pts with severe sepsis complicated with acute organ dysfunction do not fare worse with an adjusted HR of 0.979 (0.908–1.055).	Nationwide population-based retrospective cohort study in pts with severe sepsis requiring ICU admission

CI: confidence interval; DM: diabetes mellitus; HR: hazard ratio; ICU: intensive care unit; Pts: patients.

The diabetic and non-diabetic cohorts in this study were balanced at the time of first-episode ICU admission in terms of surgical conditions (*p* = 0.4554), Charlson-Deyo comorbidity index score (*p*>0.9999) and the presence of malignant neoplasm (*p* = 0.7884) (Table 1). This may simply reflects that the two cohorts were adequately sampled from the general population by this nationwide population-based study. Since the definition of diabetes mellitus in this study is well-defined, misclassification bias is not a concern. Another possible concern with cohort studies is loss to follow-up, which was not present in this study design because every subject was followed up until death as an outcome or discharge from the hospital.

In a manner that echoes a number of prior published reports concerning the reduced risk of acute respiratory failure in diabetic patients as compared with non-diabetic patients [16], [19], [31], this study confirms this finding. The relative risk of developing acute respiratory dysfunction is statistically lower at 0.96 (95% CI, 0.94–0.97). Moreover, the adjusted HR for 90-days hospital mortality in patients with acute respiratory dysfunction at 1.020 (95% CI, 0.942–1.105) shows that the overall risks are similar in the diabetic and non-diabetic groups (Fig. 3). One of the postulated mechanisms by which the rate of acute respiratory dysfunction is affected is via reduced neutrophil bactericidal activity, impaired neutrophil chemotaxis and lower leukotriene B4 production by neutrophils as well as lower levels of superoxide production, which results in the diabetic patient suffering less oxidative damage [10], [19], [32], [33], [34]. Medications such as antidiabetic agents and insulin may also contribute to this protective effect [35], [36], [37]. In a preclinical animal study, rosiglitazone, a potent agonist of the peroxisome proliferator-activated receptor (PPAR)-γ, was found to exert anti-inflammatory effects both *in vitro* and *in vivo* that significantly reduces endotoxin-induced acute lung injury in rats [37].

Interestingly, in addition to acute respiratory organ dysfunction, the diabetic cohort in this study also have a lower risk of developing hepatic (RR = 0.77, 0.63–0.93) and hematological (RR = 0.70, 0.56–0.89) organ dysfunction (Table 2). This study is the first report to pinpoint such relationships.

The only increased RR is that of acute kidney injury, however in terms of the HR for hospital mortality rate, the group with acute kidney injury was found to have a lower risk of 0.795 (0.691–0.914). After adjustment made for age group, gender, Charlson-Deyo comorbidity index, surgical condition and number of acute organ dysfunctions, the adjusted HR became non-significant (aHR = 0.871, 95% CI 0.754–1.005). Similar to this finding, Esper et al's epidemiological study indicated that subjects with diabetes and sepsis were more likely to develop acute kidney injury when compared to non-diabetics (13% *vs.* 7%, *p*<0.05) and were also less likely to develop acute respiratory failure (9% *vs.* 14%, *p*<0.05) [16].

The risk for mortality was statistically significantly increased in male patients (OR 1.3), patients aged older than 80 years (OR 1.3), patients with a higher CCI score of greater than 2 (OR 1.6 for moderate score group and 1.9 for high score group) and patients having more than one acute organ dysfunction (OR 2.5 for 2 organ dysfunction and 4.0 for >/ = 3 organ dysfunction). However, the 60–70 year age group had a lower risk, namely an OR of 0.8 (0.698–0.945). Thus diabetes mellitus status does not predict patient mortality outcome based on the present multiple logistic regression analysis.

This is the first nationwide, population-based study to examine the effect of diabetes on the outcome risk for severe sepsis requiring ICU admission. This study has a number of strengths. These include, firstly, the use of a previously validated population-based dataset that enables the complete follow-up of both cohorts with no drop-out or intentional exclusion from the final analysis. Secondly, the use of a large sample size that allows a considerable statistical advantage when detecting real differences between the diabetic and non-diabetic cohorts. Nevertheless, this study still has some limitations. In this context, the major and most obvious limitation is that some patient information, such as cigarette smoking, alcohol consumption and glycated hemoglobin HbA1c level, as well as a detailed bacteriological or fungal pathogen analysis, were not available through the administrative dataset. Through this nationwide population-based study of Taiwanese patients with severe sepsis who required ICU medical care, it was found that diabetes status does not influence the subsequent outcome with respect to either hospital mortality or length of hospital stay. Interestingly, diabetic cohorts seem to have a lower risk of developing acute respiratory dysfunction, acute hepatic dysfunction and hematological dysfunction.

## Interpretation

This is the first report utilizing a nationwide population-based follow-up study design that provides further strong evidence to refute the arguments for diabetes has negative impact on the outcome of severe sepsis. This study is also the first report to pinpoint such relationships as the diabetic cohort has a lower risk of developing acute hepatic and hematological organ dysfunction. The limitations in this study is that some patient information, such as cigarette smoking, alcohol consumption and glycated hemoglobin level, HbA1c level, as well as a detailed bacteriological or fungal pathogen analysis were not available through the administrative dataset. Future research should focus on examining the association between certain antidiabetic agent and the outcome of severe sepsis.
